# Profiling of bushmeat value chain actors in the northern sector of Ghana for targeted interventions to reduce zoonotic disease and public health risks

**DOI:** 10.3389/fvets.2026.1691095

**Published:** 2026-01-27

**Authors:** Blaise Ouattara, Cecilia Akita, Boi Kikimoto, Benjamin Obukowho Emikpe, Derrick Adu Asare, Prince Nana Takyi, Salisu Shaban, Jude Dzevela Kong

**Affiliations:** 1 Regional Food Safety and Quality Officer for Africa at the Food and Agriculture Organization of the United Nations (FAO), Accra, Ghana; 2 Department of Pathobiology, School of Veterinary Medicine, Kwame Nkrumah University of Science and Technology (KNUST), Kumasi, Ghana; 3 Department of Agricultural Science Education, University of Education, Winneba, Ghana; 4 Laboratory for Industrial and Applied Mathematic (LIAM), Department of Mathematics and Statistics, York University, Toronto, ON, Canada

**Keywords:** bushmeat trade, Ghana, profiling, public health risks, zoonotic disease

## Abstract

**Background:**

The bushmeat trade in Northern Ghana is important for rural economies and food security, but it faces challenges like poor hygiene, the risk of zoonotic diseases, and the fact that it is not sustainable. There is insufficient information regarding the principal actors, their socio-economic functions, and adherence to hygiene and food safety standards.

**Methods:**

A mixed-methods design was employed to gather data from 61 purposely selected participants involved in the bushmeat value chain in Ghana's Upper East and Upper West regions. The methods used to collect data included structured questionnaires, focus group discussions with hunters, traders, consumers, and wildlife officers, direct observations, and participatory techniques to find out how food is distributed and how safe it is to eat.

**Results:**

The study found that all the hunters were men between the ages of 25 and 50, with a majority lacking formal education (59.5%), who worked part-time mostly during the dry season and earned low monthly incomes, with over half making less than GHs100. Most of the hunters and traders had not been taught how to keep food safe. Almost all of the meat (97.3%) was processed in the bush using traditional smoking and then sold in dirty places. Seventy percent of trade was in smoked meat, and 58.8% of that was moved in baskets or bags. Hunters had low pay, a 95% drop in wildlife, and prices in the market that traders set.

**Conclusion:**

Northern Ghana's bushmeat trade is a multifaceted social, economic, and public health issue that requires targeted interventions to enhance sanitation, develop new revenue streams, and more effectively implement laws protecting wildlife. To effectively deal with these problems, all stakeholders, including local communities, government agencies, and NGOs, need to work together to find a sustainable balance between protecting biodiversity, public health, and the economic wellbeing of rural people.

## 1 Introduction

The demand for wildlife products, especially bushmeat, has gone up tremendously in the last few years. This has turned it into a profitable business that gives jobs to thousands of men, women, and young people in Sub-Saharan Africa (1). Bushmeat is a big part of the animal protein intake in many communities. In some rural areas of West Africa, it makes up as much as 84% of their protein needs (2). The growing demand for bushmeat is driven by a preference for its taste compared to domestic meat. Bushmeat also offers important nutritional value and provides essential minerals, especially during the dry season when other dietary sources may not be readily available (3).

In countries with limited resources, bushmeat hunting is both an efficient means of getting food and a way to earn income. Bushmeat is important to the social structure of many Sub-Saharan African societies because it has cultural, economic, and nutritional value (4). Even though there are laws against it, the bushmeat trade is still very important for the people who live in the forests of West and Central Africa (5, 6).

Bushmeat consumption increased in both rural and urban regions, significantly contributing to food security (7). This is especially significant in rural areas, where it functions as an essential dietary staple, providing crucial support to vulnerable households during periods of economic hardship and throughout the dry seasons (8). Nevertheless, the bushmeat trade presents significant public health risks, particularly regarding the transmission of zoonotic diseases. There are documented links between eating bushmeat and outbreaks of diseases like Ebola (specifically during the 2013 epidemic), Mpox, Marburg, and COVID-19. This indicates the risks of unregulated hunting and how it affects human health. The likelihood of disease transmission is heightened by habitat reduction and increased interactions between humans and wildlife, particularly in densely populated areas (9, 10).

Bushmeat markets are prevalent in Africa, especially in West Africa, yet their structure and distribution are not well understood. These markets collect wild animals from nearby areas, with trade patterns affecting species, volume, and price overtime (11, 12). The Food and Agriculture Organization notes that the bushmeat trade supports many livelihoods, but limited data on participants and food safety compliance make it hard to gauge zoonotic risks or needed safeguards (13).

There are knowledge gaps on the integration of the Ghana bushmeat trade within broader public health policies, particularly zoonotic disease transmission, hygiene practices, and sustainable wildlife utilization. While previous research has concentrated on consumption patterns and their effects on biodiversity, the human aspects and value-chain interactions have received limited attention (14). Addressing these issues is fundamental to evidence-based public health interventions and sustainable resource governance.

Furthermore, enhancing public awareness of human–nature interactions can be achieved through the concept of Nature Quotient (NQ), which represents a form of intelligence that fosters coexistence with nature and effective utilization of resources (15). Enhancing NQ among individuals, governmental bodies, and NGOs can create more integrated systems that connect conservation, livelihoods, and health awareness.

It is important for public health measures to work well to understand who is involved in the bush meat trade, how zoonotic diseases are found, and how to reduce risks. The study aimed to provide a detailed profile of bushmeat value chain actors in the Northern sector of Ghana, focusing on their socio-economic functions, hygiene practices, and interactions with regulatory authorities. This study frames its findings within the frameworks of NQ and systems thinking to inform culturally sensitive interventions aimed at enhancing sustainable livelihoods and mitigating public health risks.

## 2 Methods

### 2.1 Study design

The study used structured questionnaires, focus group discussions, and direct observation to collect both quantitative and qualitative data on the bushmeat trade. This approach provided demographic statistics and insights into participants' experiences and challenges in the value chain. A mixed-methods design enabled the collection of evidence from multiple sources, thereby enhancing the validity and reliability of the findings. Quantitative tools allowed for statistical profiling of actors, while qualitative methods, particularly focus group discussions, offered context on the social and cultural dimensions of bushmeat practices.

### 2.2 Study area

**Figure 1:**
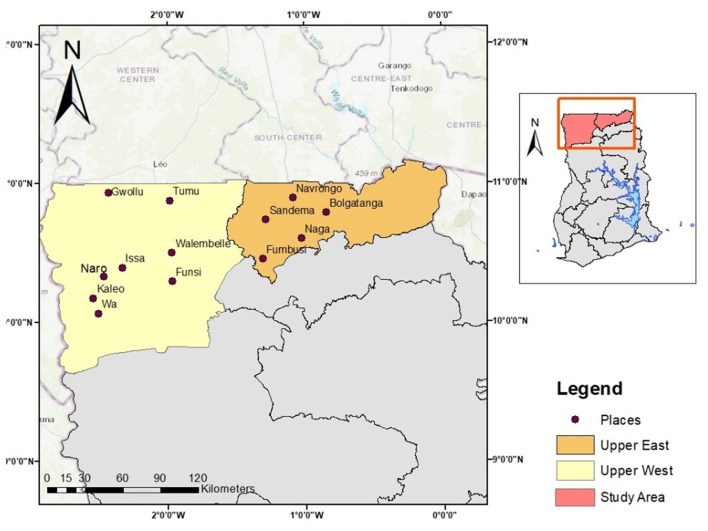
Map of the study area showing the study region and study towns.

The study was conducted in the Upper West and Upper East Regions of Ghana. The selected communities included Wa, Kaleo, Naro, Gwollu, Walembelle, Funsi, Tumu, Bolgatanga, Naga, Issa, Navrongo, Sandema, and Fumbisi as shown in Figure 1. These areas were selected based on their active bushmeat trade network, involving hunters, traders, and wildlife officers. Hunters sell meat to traders, who then process it and send it to urban and rural markets through systems that are usually based on family ties. It is possible to buy northern bushmeat in southern Ghana, which shows how far the trade reaches. While these networks are an important way for people in rural areas to make a living and for people in cities to get food, they also pose major health and environmental risks.

### 2.3 Study population and sampling

The study population consisted of 37 bushmeat hunters, 17 traders, and 7 Wildlife Technical Officers from specific northern regions of Ghana. Participants were chosen based on their active engagement in the bushmeat value chain, encompassing roles in hunting, trading, and regulatory oversight. Sixty one questionnaires were distributed, and 3 mixed focus group discussions were also conducted among hunters, traders and regulators. Each group had a minimum of 12 hunters, 5 traders and 2 regulators in order to get a wide range of experiences and practices in the field.

### 2.4 Data collection instrument

To investigate the bushmeat trade, various methods of collecting data were used. Structured questionnaires for hunters, traders, and wildlife technical officers yielded quantitative data on demographics, practices, and perceptions. Observations evaluated hygiene standards and trading conditions, whereas focus group discussions provided qualitative insights into challenges, beliefs, and coping mechanisms.

### 2.5 Data collection procedure

A team of two FAO staff and a veterinary officer from the Kwame Nkrumah University of Science and Technology carried out the data collection across the selected communities. Participants were purposively selected based on their active involvement in the bushmeat value chain. Primary data were collected through questionnaires, focus group discussions, and field observations, scheduled during local market days and community visits to ensure access to participants in their natural settings and during peak business activities. Each session lasted for 15–25 min, allowing for adequate engagement with participants. The focus group discussions were conducted in local dialects, with interpreters assisting to facilitate participation and accurate translation of responses. The team also assessed hygiene practices, meat processing methods, and market sanitation. All responses and observations were recorded and cross-checked to ensure data quality.

### 2.6 Ethical considerations

Ethical standards were maintained: verbal consent was given by all participants after study details were explained, participation was voluntary, and withdrawal was allowed at any time. Identifiable data were removed to ensure confidentiality, and results were reported in aggregate.

### 2.7 Data analysis

The collected data were analyzed using descriptive statistical methods. Quantitative responses were summarized with frequencies, percentages, and graphs to identify trends and patterns among the actors. Qualitative insights from interviews and focus group discussions were analyzed with R version 4.4.1 (27). A datasheet was set in Microsoft Excel, which transformed the interview transcript into codes. Each response provided by participants was interpreted and transformed to corresponding code. Transcripts were coded to identify recurring concepts, which were progressively grouped into analytical codes and higher-level themes reflecting practices among bushmeat Hunters, traders, and Wildlife Technical Officers. Results were clearly presented in graphs and tables.

## 3 Results

### 3.1 Key stakeholders in the bushmeat value chain

Key stakeholders in the bushmeat value chain are hunters, traders, individual consumers, and the Wildlife/Forestry Commission. Hunters supply bushmeat to traders and consumers, while traders process it in the markets. The Wildlife/Forestry Commission oversees forest conservation and handles permits and licenses for both traders and hunters.

### 3.2 Hunters

**Figure 2:**
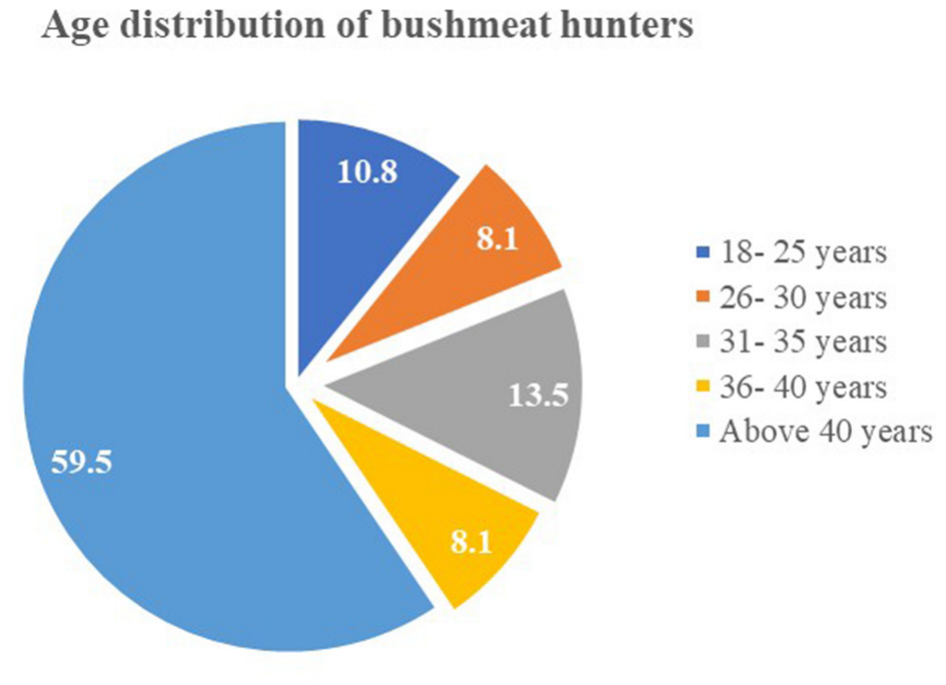
Age distribution of bushmeat hunters.

**Figure 3:**
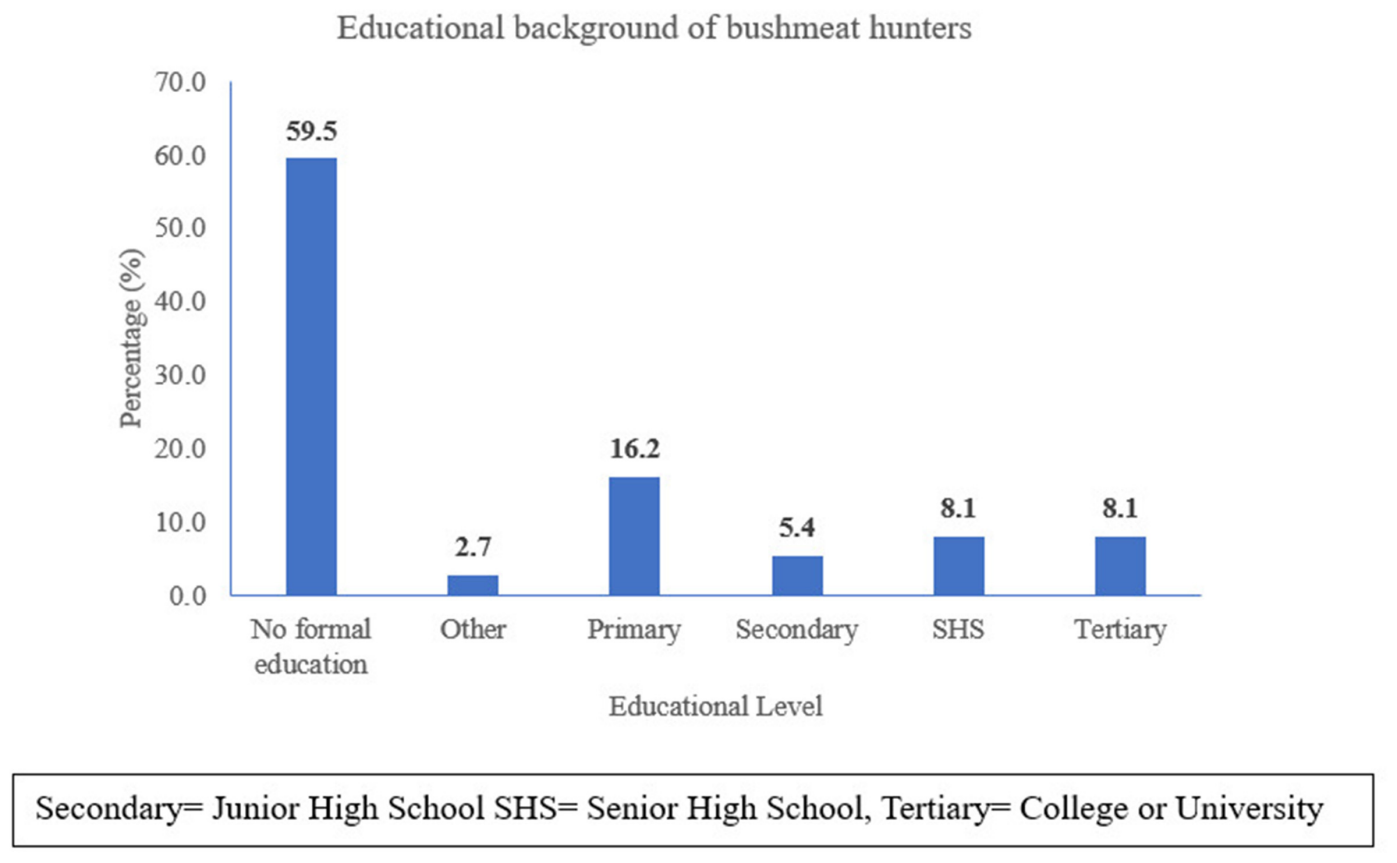
Educational background of bushmeat hunters.

The hunters in the bushmeat value chain are predominantly male, with 100% of the participants being men, all of whom are married. The majority of hunters (59.5%) are aged above 40 years, reflecting the age demographic of individuals involved in bushmeat hunting in the region and the same proportion have no formal education, highlighting a gap in formal schooling (Figures 2 and 3). These demographic factors suggest that bushmeat hunting is often a long-standing livelihood activity, typically passed down through generations, and primarily serves as a supplementary income during the off-season, particularly in rural and agricultural communities.

#### 3.2.1 Bushmeat hunting practices, processing, and hygienic practices

Bushmeat hunting primarily occurred during the non-farming or dry season (Table 1). According to the hunters, religious beliefs and practices led them to perform halal slaughter on animals that were shot in the bush (97.3%). They also reported using fire from firewood to smoke the meat (94.6%) in the bush to prevent spoilage before transporting it to the village for further sales. The meat is typically sold directly to individuals or bushmeat traders who may have partially or fully sponsored the hunters, rather than being sold at markets. Most hunters (83.8%) indicated they had not received any training in good hygienic practices related to bushmeat handling.

**Table 1:** Bushmeat hunting, processing, and training on good hygienic practices.

**Variable**	**Response**	**Frequency (*n*)**	**Percentage (%)**
Hunting period	Dry Season	37	100.0
Mode of the slaughter of bushmeat	At home	1	2.7
	In the bush	36	97.3
Method of processing bushmeat	Drying	1	2.7
	Salting and Smoking	1	2.7
	Smoking	35	94.6
Mode of smoking bushmeat	Charcoal	1	2.7
	Firewood	35	94.6
Received training on good hygienic practices	Yes	6	16.2
	No	31	83.8

**Figure 4:**
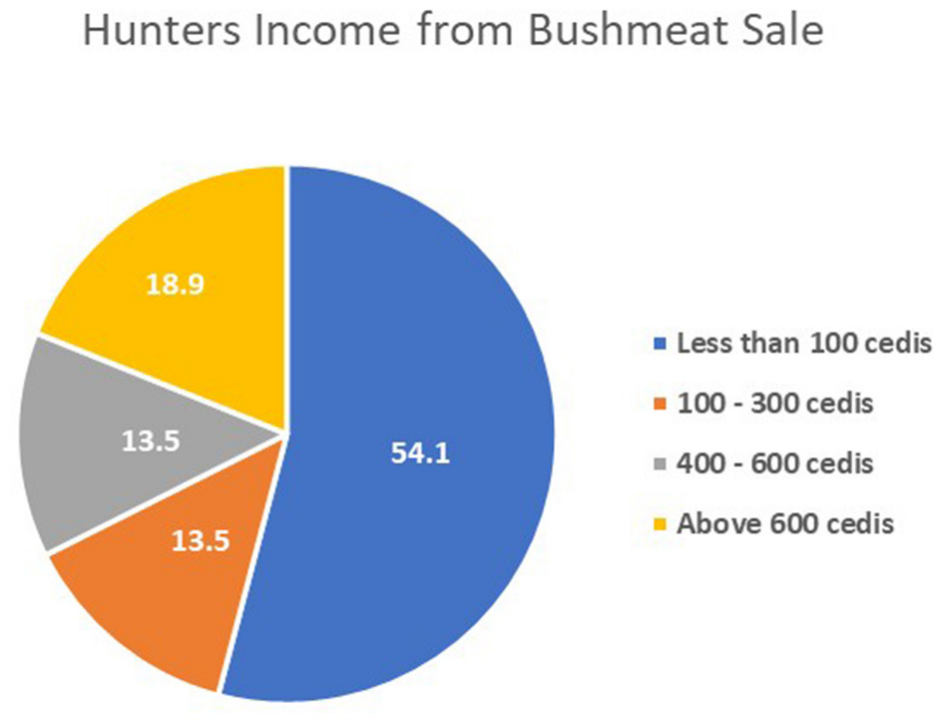
Hunters' monthly income generated from the bushmeat trade.

**Figure 5:**
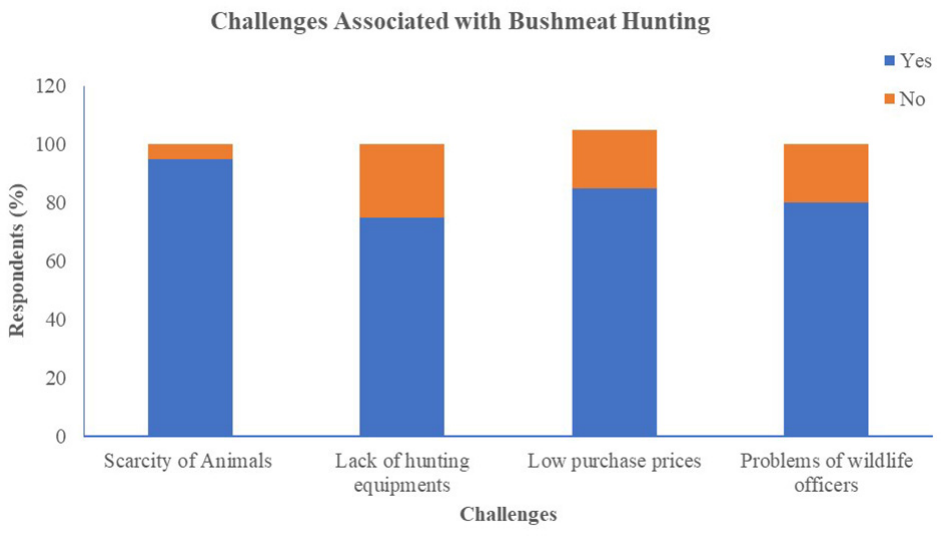
Hunter's challenges associated with hunting bushmeat.

The questionnaire showed that hunting yields low profit, with 54.1% of hunters earning under Ghs100 per month from bushmeat sale (Figure 4). Hunters attributed this to several issues: 95% cited declining wildlife population; 85 said traders set low purchase prices; 80% mentioned interference from wildlife officers; and 75% faced shortages of essential hunting equipment (Figure 5).

#### 3.2.2 Cultural issues associated with hunting

The focus group discussions with hunters and related participants revealed that hunting is linked to mystic beliefs, including midnight interactions with supernatural forces. Hunters report encountering ghosts and strange beings, highlighting the significant risks they face in their work.

### 3.3 Bushmeat traders and mode of sales and preservation

#### 3.3.1 Traders

In the bushmeat value chain, traders play a vital role by acting as intermediaries between hunters and consumers. They are responsible for processing, preserving, and distributing bushmeat in local markets. This group is predominantly made up of women (88.2%), reflecting gendered roles in the trade, with men often involved in hunting and women taking on the tasks of retail and market-based sales (Table 2). Most did not have formal education (76.5%), and the same proportion participated in bushmeat trading on a part-time basis. Though they are critical to the commercialization of bushmeat, they face numerous challenges, from limited education and lack of food safety training to economic pressures and poor working conditions.

**Table 2:** Demography of bushmeat marketers, state of sale and convey of bushmeat to the market.

**Variable**	**Categories**	**Frequency (*n*)**	**Percentage (%)**
Gender	Male	2	11.8
	Female	15	88.2
Education level	No formal education	13	76.5
	Primary	1	5.9
	JHS	3	17.6
Type of bushmeat trade	Part-time	13	76.5
	Full-time	4	23.5
State of sale of bushmeat	Smoked	12	70.6
	Fresh & smoked	5	29.4
Mode of convey of bushmeat to market	Baskets	5	29.4
	Sacks & Baskets	10	58.8
	Sacks	2	11.8
Mode of displaying bushmeat at market	On tables	3	17.6
	On sacks	4	23.5
	On trays	10	58.8
Mode of preservation of bushmeat	Smoking (under low heat)	17	100

#### 3.3.2 Mode of sales and preservation

**Figure 6:**
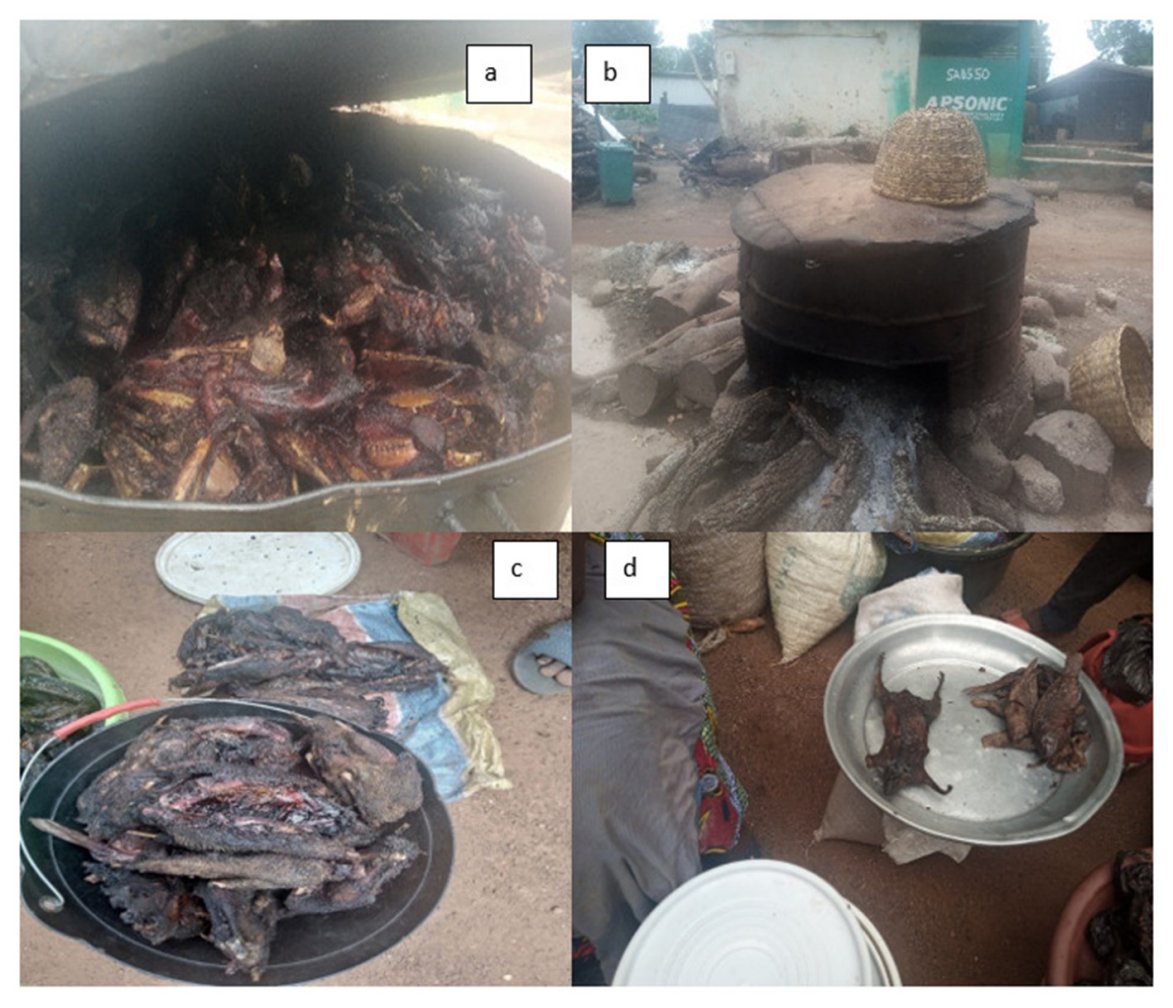
**(a, b)** Method of smoking bushmeat as well as for preservation. Open display of bushmeat on trays **(c)**, and sacks (on the floor) **(d)**.

Regarding bushmeat sales, 70.6% of traders smoked bushmeat. The majority transport these products in sacks and baskets (58.8%), while a smaller proportion uses only baskets (29.4%). Observations indicate that bushmeat is predominantly displayed openly on trays similar to the proportion of products in sacks and baskets, followed by on sacks (23.5%) and on tables (17.6%), which may expose the meat to environmental contaminants such as flies, dust and other infectious pathogens, which may have zoonotic implications. The preservation technique employed was exclusively smoking (100%) as shown in Table 2, which was distinctive amongst the women, utilizing large open-topped drums as illustrated in Figures 6a, b.

#### 3.3.3 Issues associated with sales of bushmeats

Observations and focus group discussions showed that bushmeat traders often also sold fish, especially when bushmeat was scarce. Traders who possessed bushmeat hid it to avoid arrest. Both meat and the fish were displayed uncovered on metal trays Figures 6c, d, attracting many flies, dust and other infectious pathogens, which may have zoonotic implications.

### 3.4 Wildlife/forestry commission technical officers

All technical staff were male, aged 26 and above, with 85.7% having completed tertiary education. Their hunting experience ranged from 3.5 to 39 years, averaging 11.7 ± 13.6 years (Table 3).

**Table 3:** Demography of wildlife technical staff.

**Variables**	**Categories**	**Frequency**	**Percentage (%)**
Age	26–30 years	2	28.6
	31–35 years	1	14.3
	36–40 years	1	14.3
	Above 40 years	3	42.9
Educational Levels	SHS	1	14.3
	Tertiary	6	85.7
	**Mean**	**Maximum**	**Minimum**
Length of work experience	11.7 ± 13.6 years	39 years	3.5 years

**Figure 7:**
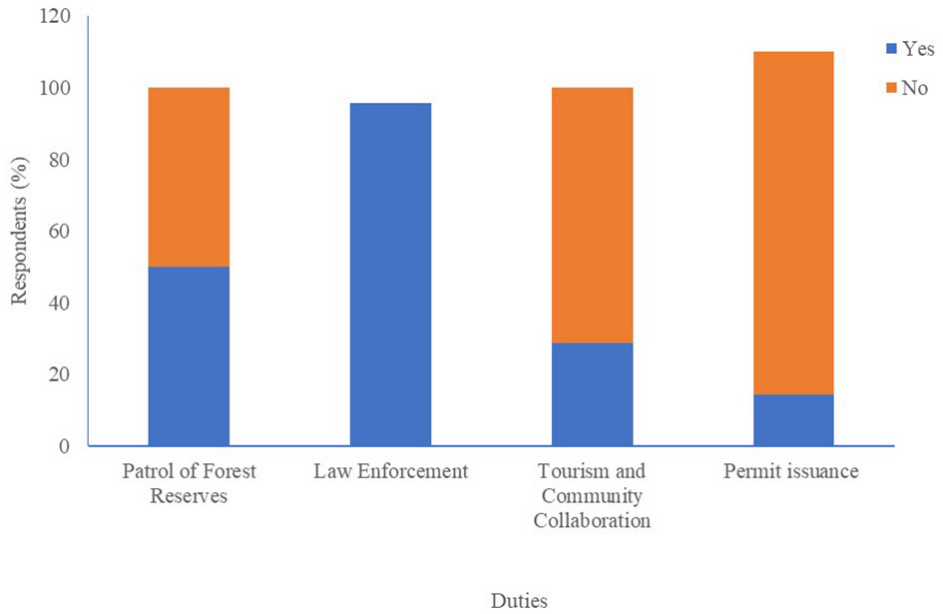
Responsibilities of wildlife technical officers.

Wildlife technical officers are primarily responsible for enforcing laws against illegal hunting of endangered species (100%). Additionally, they patrol forest reserves (50%), guide tourists in wildlife forest reserves (28.6%), and handle permits and licenses for both hunters and traders (14.3%) (Figure 7).

Wildlife technical officers encounter several challenges in their work, as outlined in Table 4. These include limited staff numbers, lack of motivation, inadequate working conditions, insufficient transportation for apprehending hunters, poor communication devices, and incidents involving conflicts with hunters, among other issues.

**Table 4:** Challenges faced by technical officers.

Poor conditions of services, old weapons
Low number of staff, lack of staff motivation
No means of transporting arrested hunters
Poor communication devices
Inadequate training
Attacks from the hunters
Harsh weather conditions

Comparatively, traders are contributing more to the unhygienic practices occasioned by the display of meat and fish uncovered on metal trays that can cause post-processing contamination.

## 4 Discussion

This study investigates the patterns of the bushmeat trade in the Northern Belt of Ghana, describing its socio-economic, public health, and ecological implications. Profiling key actors, including hunters, traders, and technical officers, reveals a complex interplay of economic survival, cultural practices, and public health issues. The bushmeat trade is a way for people to get meals and earn during the off-season for farming. This is especially true for male hunters, who often do the trade part-time. This trend is similar to what has been identified in other West African countries, such as Nigeria and Ivory Coast, where bushmeat is very important to the economy and culture (16, 17).

The Northern Belt of Ghana has been identified as a region of zoonotic risk due to the human-wildlife interactions, seasonal wildlife movement, and zoonotic infections reported in neighboring countries (10, 14). Profiling bushmeat value chain actors provides an evidence base that directly supports Ghana's One Health agenda, the National One Health Policy Framework, and zoonoses control strategies by identifying high-risk practices, priority actor groups, and intervention points for surveillance, risk communication, and preventive action (18).

The findings indicate low levels of Nature Quotient (NQ) among study participants, as most actors demonstrated limited knowledge of zoonotic risks and environmentally sustainable wildlife practices. NQ, as described by Vuong and Nguyen (15), embodies the principle of interacting with nature and engaging in informed decision-making that promotes both human and environmental wellbeing. The development of NQ has the potential to enhance control of the bushmeat trade and improve health awareness.

Given the limited population for formal education and media exposure in the Northern part of Ghana, initiatives to improve NQ can be optimized through culturally appropriate communication channels. Proverbs, fables, and legends present in community discourse can serve as effective means for conveying hygiene and conservation practices (19, 20). The integration of public health messages into cultural pathways complements systems-thinking approaches that merge local knowledge with contemporary health and conservation efforts.

Zoonoses prevention, hygiene, and wildlife conservation messages should be embedded into existing community platforms, traditional gatherings, and market associations, alongside targeted training of wildlife officers, hunters, and traders on safe handling and reporting practices; this approach strengthens Nature Quotient (NQ) by aligning local cultural knowledge with public health awareness, while simultaneously improving early disease detection, surveillance linkages, and food safety compliance along the bushmeat value chain (16, 19).

The study reveals a gendered bushmeat value chain: men primarily participate in hunting, while women are mainly tasked with trading, retailing, and processing. This division of labor is important for creating targeted health and food safety programs because different approaches may be needed for men and women. Also, the fact that hunters and traders have low levels of education makes things harder, which is in line with studies in Nigeria that show that higher education leads to safer practices when it comes to zoonotic risks (17, 21).

Hygienic practices along the bushmeat value chain are poor. Most hunters process meat in the bush using traditional methods like halal slaughter, boiling, drying, and smoking. These methods are based on cultural and religious beliefs (22). But 83.8% of hunters and 76.5% of traders had never received any formal training in how to handle meat safely, which is a big risk to public health. People often put meat on trays and sacks without any covering in markets. This makes it easy for flies, dust, and other pathogens to get on it. Similar issues exist in Nigeria and Sierra Leone, where bushmeat workers do not wash their hands or wear personal protective equipment (PPE), which is also a cause for concern (17, 23, 24). Without formal training and proper infrastructure, the risk of zoonotic disease transmission increases, emphasizing the need for food safety education. It was observed that practices related to meat processing, handling, transport, and market display, as described, could be linked to potential public health and zoonotic risk pathways, as often seen in most West African countries.

Economic conditions also contribute to these risks. More than half of the hunters reported earning less than GHs100 per month from bushmeat sales, which is attributed to factors such as reduced wildlife populations, limited bargaining power with traders, and enforcement pressure from wildlife officers. This economic situation often leads individuals to continue participating in the trade, despite associated health risks. These patterns are consistent with findings of Nielsen et al. (25), which identified economic necessity, inheritance of the trade, and unemployment as the main reasons for continued engagement in bushmeat hunting, regardless of awareness about zoonotic risks.

Cultural factors contribute to the perpetuation of hunting practices. In some regions, hunting is both a livelihood and a spiritual tradition, with rituals and beliefs shaping local practices. These customs can conflict with modern conservation and food safety rules. Effective intervention must balance cultural respect with the introduction of safer, sustainable methods.

Wildlife Technical Officers regulate the bushmeat trade, enforce hunting restrictions, and issue permits. However, their effectiveness is limited by staffing shortages, equipment deficits, transportation issues, and safety concerns. These challenges hinder both law enforcement and zoonotic disease surveillance, as seen in Zimbabwe and Nigeria, where resource constraints weaken wildlife protection efforts (17, 26). This may lead to trafficking in endangered species and their products.

Zoonotic disease outbreaks like mpox, Ebola, and COVID-19 highlight the significant public health risks of the bushmeat trade, as clearly related to meat processing, handling, transport, and market display, as described in this study. The study calls for a holistic One Health approach that combines public health education, sustainable livelihood, stronger wildlife protection enforcement, and improved veterinary and food safety services. Interventions should be culturally sensitive and adapted to the socio-economic conditions of those involved (16), ensuring that both human health and environmental conservation are addressed.

The bushmeat trade in Northern Ghana poses complex economic, cultural, and public health challenges. Addressing these requires a systems-thinking approach that supports community needs, sustainable wildlife use, and public health. Interventions should improve hygiene and food safety practices, provide alternative livelihoods to reduce reliance on bushmeat hunting, and boost wildlife law enforcement. Effective solutions depend on cooperation between government agencies, non-governmental organizations, and local communities to balance social, health, and conservation priorities.

## 5 Limitations

The sample was restricted to two regions in the north and might not reflect the complete variability of Ghana's bushmeat trade. Secondly, some of the responses were based on self-reporting, which could be affected by social desirability bias, especially concerning hygiene and compliance issues with regulations. Thirdly, testing to validate the presence of zoonotic pathogens in the bushmeat samples was not undertaken in the study. Lastly, only a small number of participants responded to the questionnaire. Despite these limitations, the mixed-method design provided findings on socio-economic and behavioral factors, forming a basis for further research.

## 6 Conclusion

In Northern Ghana, the bushmeat trade is essential to local livelihoods, especially during the off-season. However, it also presents risks of zoonotic diseases, poor hygiene, and unstable economic conditions. A large amount of meat is processed and sold in unhygienic conditions, and many hunters and traders lack food safety training. Financial difficulties affect hunters, and wildlife officers lack the resources necessary to effectively regulate the sector. The results point to the necessity of interventions that take into account the bushmeat trade's socioeconomic and public health components. These interventions ought to be culturally relevant, context-specific, and take into account the opinions of different stakeholders, such as hunters, traders, governmental organizations, and non-governmental organizations.

## 7 Recommendation

A comprehensive strategy is needed to address the issues surrounding the bushmeat trade in Northern Ghana. Since many hunters and traders are not trained in food safety, it is imperative to improve hygiene practices. Consumer meat safety will be ensured and health risks related to zoonotic diseases will be reduced with the implementation of easily accessible, hands-on hygiene programs. Diversification of livelihoods is equally important. Communities can lessen their dependency on bushmeat and attain financial stability during off-seasons by offering alternate revenue streams like agroforestry or small livestock farming. Additionally, this would reduce the strain that unsustainable hunting methods place on the environment and human health. Enhancing market infrastructure is yet another important factor. Public health standards will be raised and contamination risks reduced with the implementation of covered stalls, clean water access, and basic hygiene supplies like gloves for vendors. In order to promote sustainable hunting methods and boost compliance, wildlife law enforcement must be strengthened in conjunction with public awareness initiatives. To balance public health, conservation, and economic needs, government agencies, non-governmental organizations, and local communities should work together.

## Data Availability

The original contributions presented in the study are included in the article/supplementary material, further inquiries can be directed to the corresponding author.
